# Is monkeypox another reemerging viral zoonosis with many animal hosts yet to be defined?

**DOI:** 10.1080/01652176.2022.2088881

**Published:** 2022-06-19

**Authors:** D. Katterine Bonilla-Aldana, Alfonso J. Rodriguez-Morales

**Affiliations:** a Faculty of Medicine, Institución Universitaria Vision de las Américas, Pereira, Risaralda, Colombia; b Universidad Científica del Sur, Lima, Perú; c Grupo de Investigación Biomedicina, Faculty of Medicine, Fundación Universitaria Autónoma de las Américas, Pereira, Risaralda, Colombia; d Latin American network on MOnkeypox VIrus research (LAMOVI), Pereira, Risaralda, Colombia

Again, the world is witnessing a disease caused by a member of an old group of viruses that may go into the spillover from animal species to humans (Bezerra-Santos et al. 2021). In 2022, the monkeypox virus (MPXV), an Orthopoxvirus (family Poxviridae), is causing a multi-country outbreak in almost all the continents outside Africa, which has been endemic since its discovery in humans in 1970 in the Democratic Republic of Congo (León-Figueroa et al. 2022).

Up to June 15, 2022, 2027 cases of human MPXV infections have been confirmed in more than 35 countries across Europe, North America, Latin America, the Middle East, and the Pacific regions (WHO 2022). Besides the initial description of the virus, among cynomolgus monkeys (*Macaca fascicularis*), in Denmark in the summer of 1958 (Magnus et al. 1959), and the 2003 United States of America (USA) outbreak associated with the illegal trade of prairie dogs (*Cynomys ludovicianus*) from Africa (Reed et al. 2004), both related to animal-to-human transmission; no animals have been implicated in the transmission or description of human cases outside Africa, that have been reported especially after the 2017-2018 Nigeria outbreak with imported cases mainly in the United Kingdom (UK), Singapore and USA (Rodríguez-Morales et al. 2022).

Before the current outbreak of MPXV, it was well-known that this is a zoonotic disease, with its pathogen found in multiple mammals in Africa, especially rodents, including *Cynomys* spp., but also the Gambian pouched rats (*Cricetomys gambianus*) (which was implicated in the 2003 US outbreak, as the imported prairie dogs were previously housed with these rats) (Falendysz et al. 2015, Parker and Buller 2013). In addition, species of *Funisciurus* (squirrels)*, Heliosciurus* (squirrels)*, Oenomys* (rufous-nosed rats)*, Graphiurus* (African dormice)*, Cricetomys* have been seropositive to MPXV (Doty et al. 2017). Other mammals, such as *Sus scrofa* (domestic pig) and the *Macaca mulatta* (rhesus macaque), are also susceptible animals to MPXV infection (Hutin et al. 2001, Schmitt et al. 2014). But many others would be implicated. MPXV has been already considered a neglected pathogen (Cohen 2022), and a lack of research on it has been recently demonstrated (Rodríguez-Morales et al. 2022).

From the animal perspective, many questions must be addressed in addition to a more straightforward definition of the broad spectrum of susceptible hosts, and natural and experimental infections, among others. But even its origin. Monkeypox is a disease described in Europe, not in Africa. MPXV was identified in 1958 in Copenhagen, Denmark, during two outbreaks in cynomolgus monkeys used for polio vaccine research (Magnus et al. 1959, Parker and Buller 2013). Those monkeys were imported from Singapore. Those animals developed vesiculopustular skin eruptions observed over the entire trunk, tail, face, limbs, palms of the hands and feet soles of the feet (Bezerra-Santos et al. 2021, WHO 2022). Does the MPXV come from Singapore? By the way, Singapore has reported imported cases of MPXV (Yong et al. 2020). After 1958, other reports of MPXV among colonies of captive monkeys were also described in the USA (1959 and 1962) and Rotterdam Zoo, the Netherlands (1964) (Parker and Buller 2013).

In the article of von Magnus (1959), a previous pox-like disease outbreak in monkeys is discussed (Magnus et al. 1959). During a mild smallpox epidemic among human natives of Alto Uruguay, Brazil, *Mycetes seniculus* and *Cebus capucinus* monkeys developed typical pustules and died in large numbers. However, the virus was not identified nor recovered from those animals, raising many questions (Bleyer 1922). That raises the question of the monkeys' susceptibility to smallpox. Even more, other non-human primates (NHP), such as the African gorillas (*Gorilla gorilla*), chimpanzees (*Pan troglodytes*), the Asian gibbon (*Hylobates lar*), the South American squirrel monkeys (*Saimiri sciureus*), the African owl-faced monkeys (*Cercopithecus hamlyni*), the mangabey (*Cercocebus atys*) and the South American marmoset (Family Callitrichidae) have also been affected by MPXV, as reported during the 1964 Rotterdam zoo outbreak (Parker and Buller 2013, Radonić et al. 2014), in addition to other NHP, e.g. the baboon (Heberling and Kalter 1971).

So far, it is unclear when or how the spillover of MPXV occurred from monkeys to rodents or if these small mammals were previously infected. In any case, in 1979, apparently, the first study of natural infection among them was held in Zaire, today the Democratic Republic of Congo. This country has reported the highest number of human monkeypox cases (Khodakevich et al. 1986). In those studies, *F. anerythrus* (Thomas's rope squirrel or redless tree squirrel) was found positive. That was confirmed later by other studies detecting MPXV in that squirrel species. Some authors currently suggest that several rodents besides squirrels (families Muridae and Nesomyidae) and shrews (order Eulipotyphla) are potential monkeypox virus reservoirs (Marien et al. 2021). Indeed, more studies are needed to understand the risks of human-animals contact during the ongoing multi-country outbreak of MPXV and if other rodents and even closer mammals (e.g. domestic dogs and cats) would be at risk of being infected, with their potential consequences. As some studies suggest, this scenario would be even more complex for zoonotic transmission due to climate change. Models employed in African countries would be useful for identifying areas where environmental conditions may become more suitable for human MPX; targeting candidate reservoir species for future screening efforts; and prioritizing regions for future MPX surveillance efforts (Thomassen et al. 2013).

Indeed, long-term monitoring of emerging and reemerging viral zoonotic diseases is crucial for MPXV and other pathogens (Chakraborty et al. 2022). OneHealth approaches are urgently needed. Multi and interdisciplinary work around these biological threats is also necessary. And, albeit no less important, it is also relevant, in addition, to performing it in humans, to develop rapid assessments of the risk related to animal-human and human-animal interphases in the context of the reemergence of MPXV in the world.

## Author contributions

AJRM and DKBA formulated the letter. Next, all authors critically reviewed the manuscript for relevant intellectual content. Finally, all authors have read and approved the final version of the manuscript.
